# Self-management interventions in primary care practices in France between 2010 and 2022: a descriptive national study

**DOI:** 10.1017/S1463423626100929

**Published:** 2026-03-06

**Authors:** Emmanuel Allory, Marion Delaurens, Ronan Garlantézec, Rémi Gagnayre

**Affiliations:** 1 Department of General Practice, Univ Rennes, 2, Avenue du Pr Léon Bernard, RENNES Cedex, 35043, France; 2 CHU Rennes, Inserm CIC 1414 (Centre d’Investigation Clinique), Rennes, 35000, France; 3 LEPS (Laboratory Educations and Health Promotion), University of Sorbonne Paris Nord, UR 3412, Villetaneuse, F-93430, France; 4 Univ Angers, POPS, SFR ICAT, F-49000 Angers, France; 5 Department of Public Health, Pontchaillou Hospital, University Hospital, 35033 Rennes, France; 6 CHU Rennes, Univ Rennes, Inserm, EHESP, Irset (Institut de recherche en santé, environnement et travail) - UMR_S 1085, F-35000, Rennes, France

**Keywords:** Group practice, multimorbidity, patient participation, primary health care, self-management

## Abstract

**Aim:**

Our objective was to describe the self-management intervention (SMI) programmes carried out in primary care practices (PCPs) in France between 2010 and 2022.

**Background:**

SMIs are included in the recommendations for chronic disease management, but access remains inequal. Primary care has been identified as a favourable setting for their development.

**Methods:**

In partnership with the French Ministry of Health Office of Non-Communicable Diseases, we contacted all Regional Health Agencies (*n* = 18) to collect the following information from the self-management programme forms: year of authorization/declaration, SMI type, administrative structure, self-management and coordination team, and programme content.

**Findings:**

At the 13 participating Regional Health Agencies, we identified 4,922 SMI programmes among which 18% (*n* = 889) were developed in primary care settings and 5.5% (*n* = 271) in PCPs. Among the 127 forms on SMI programmes at PCPs (2.6%), multi-professional PCPs (57.5%, *n* = 73) and healthcare centres (25.9%, *n* = 33) were the most represented. All programmes had a coordinator (mostly general practitioners, 34.7%, *n* = 24) among whom 69.7% (*n* = 69) were trained in coordination. The self-management team included a mean of 8.1 (5.5) primary care providers. The main themes were diabetes (34.6%, *n* = 44), diabetes and cardiovascular diseases (15.6%, *n* = 20), and cardiovascular disease (10.2%, *n* = 13). In France, self-management programmes in PCPs are still rare, lack a multimorbidity approach, and are carried out mainly in PCPs with inter-professional collaboration. A qualitative study to identify the barriers and levers to SMI programmes in PCPs may be particularly relevant.

## Introduction

In Europe, the burden of chronic conditions on the healthcare systems is enormous and will continue to grow. For instance, in 2022, the percentage of ≥65-year-old people with two chronic conditions was higher than 30% (OECD and European Union, 2022; Hacker, 2024). The healthcare systems are inadequately and insufficiently prepared to manage such burden (Holman 2020; Kabir *et al.*, 2022). To improve their readiness, care delivery must be reorganized (OECD and European Union, 2022). One of the most important dimensions to consider is the development of a greater place for self-management interventions (SMI) (Straub and Thekkekandam, 2023). World Health Organization (WHO) Europe defines SMIs as supportive interventions systematically provided by healthcare staff, peers, or lay persons to increase the patients’ skills and confidence in their ability to manage long-term conditions (WHO European Region, 2024). Evidence from the literature shows that SMI programmes can improve clinical outcomes, patient-reported measures, and the quality of life of people living with chronic conditions (Tsokani *et al.*, 2023). To improve access to these programmes, primary care has a unique role to play because it can reduce disparities across the population by ensuring equal access to services (Dineen-Griffin *et al.*, 2019; WHO European Region, 2024). Therefore, SMI development in primary care is encouraged, especially in France (Haut conseil pour l’avenir de l’assurance maladie, 2022).

In Europe, SMIs for chronic diseases were introduced in the 2000s. Many healthcare systems offer different SMI programmes, mainly by health care providers (HCPs) including general practitioners (GPs) and more frequently trained nurses (Nolte *et al.*, 2014). Since 2007, in France, the National Health Authority (Haute Autorité de Santé; HAS) considers SMI accessibility a priority. These interventions need to be structured according to the HAS guidelines and to be authorized by the Regional Health Agency (Agence Régionale de Santé, ARS) (Haute Autorité de Santé, 2007). SMIs can be performed in different settings, including by telephone and online (e.g. the ‘Sophia’ programme developed by the French health insurance fund), at hospitals or in education centres, which are located in the main city of each French region, and in primary care practices (PCP). For all these programmes, which are free of charge, each SMI team must complete a structured administrative form to provide information on the hosting structure, type of self-management, content, team, coordination, and local partners. This form is analysed using some quality criteria by the ARS and validated for four years. Since 2021, a less restrictive declaration procedure has been introduced to improve SMI development.

In 2013, the qualitative review by Elissen *et al*., in 13 European countries, showed that SMI remains relatively underdeveloped (Elissen *et al.*, 2013). In 2016, Schäfer *et al.* highlighted the decreased involvement of European GPs in preventive activities between 1993 and 2012 (Schäfer *et al.*, 2016). In France, using data from three national agency reports, Clet *et al.* showed that SMI development in primary care is hindered particularly by the difficulties to integrate a collective, long-term dimension into preventive clinical practices (Clet *et al.*, 2024). However, in the last few years, more and more PCPs have been developed in France (Bahiaoui, 2024). Thanks to their organization with more collaboration among the implicated professionals, they can address the lack of SMIs and improve the quality of care for people with chronic diseases (Cret *et al.*, 2022).

Most SMI approaches in Europe involve some form of delivery system design, but the nature of the strategies varies, and more data need to be collected (Nolte *et al.*, 2014). Few data are available on SMI practice at the national level, particularly in primary care (Boerma *et al.*, 2015). Yet, to improve their development in healthcare systems, particularly in primary care, it is important (i) to describe the model of SMI practices in primary care; (ii) to identify strengths and areas of improvement; and (iii) to inform decision makers and HCPs for policy and practice improvement.

Therefore, the main objective of our study was to describe the SMI programmes at PCPs nationwide in France from 2010 to 2022.

## Methods

This descriptive retrospective study focused on the SMI programmes authorized/declared by/to the 18 ARS of France between 2010 and 2022. This period was chosen because in 2010 a national decree was published in France that lists the specifications that must be met by each SMI programme. The limit date of 2022 was selected because data collection/analysis started at the beginning of 2023.

In partnership with the French Ministry of Health Chronic Disease department, in January 2023, the self-management mission managers of each ARS were contacted by email and asked to participate in this study. Among them, 15 managers (3 refused to participate) attended an online meeting in March 2023 where the study protocol was explained. After one additional refusal to participate, individual interviews were organized with each of the 14 self-management mission managers to explain in details the study and to list the data and documents that needed to be collected. As one ARS did not have any data for the study, the analysis concerned data from 13 ARS that covered 60,059,521 million French inhabitants (87.8% of the French population).

First, from the administrative SMI files stored at the ARS, the following data were extracted: SMI location (hospital or in primary care), type (group, personalized intervention), and administrative structure responsible of the programme. Specifically, for programmes in PCPs without a specific coordinator for the administrative/financial aspects, local administrative structures can help to perform these duties, for instance, through the Coordination Support Mechanism (dispositif d’appui à la coordination).

After identification of all SMI programmes carried out at PCPs, the set of specifications of the programmes authorized/validated by their regional ARS were analysed. These data were obtained from the SI-ETP website (https://si-etp.ars.sante.fr/ ), an ARS reference site that lists all SMI programmes in France. An access to the website was created specifically for the study. When several files were available for one programme, the most recent file in the system was selected. According to Appendix 2 of the 2010 French decree, these specifications are required for the SMI programme authorization application (Supplementary file 1). The absence of a standardized authorization/declaration justified the programme exclusion from the study.

### Statistical analysis

An Excel dataset was created, and the categories of the extracted data were determined on the basis of a literature review (Allory *et al.*, 2024) and exchanges among all authors. They included: PCP type, territory type according to the French National Institute of Statistics and Economics Studies (Institut national de la statistique et des études économiques, Insee) as well as SMI programme duration, coordination, content, funding bodies, description of the team and its local partners (defined as healthcare actors working with the SMI team).

The descriptive statistical analysis of the collected data was carried out using the Stata® software. Qualitative variables were described by numbers and percentages, and quantitative variables by means and standard deviation (SD).

### Ethical issues

The Rennes University Hospital Ethics Committee approved the study (approval number 23.51 of 28 April 2023; Supplementary file 2). The project complied with the reference methodology MR-004 defined by the French committee on personal data protection (Commission Nationale Informatique et Libertés, CNIL) and with the European General Data Protection Regulation. The study protocol was registered in Clinical trials® under the number NCT05950451.

## Results

At the 13 ARS that participated in the study, 4922 SMI programmes were identified (Table 1) among which 82% were at hospitals. The mean period of authorization was 6.6 years for SMIs in primary care settings. Among the 889 programmes in primary care settings, 271 were performed at PCPs, and 71.1% of these interventions were administratively supported by the PCP. Then, 144 SMI programmes were excluded for different reasons: the ARS did not give the files due to the work required (*n* = 115 programmes from three ARS); files not found by the ARS (*n* = 21); different programmes linked to only one file (*n* = 5); and absence of standardized authorization/declaration file (*n* = 3). Programmes excluded for these three last reasons were distributed equally among ARS. Therefore, the analysis focused on the 127 SMI programmes carried out in PCPs for which the authorization and/or declaration files could be accessible (Table 2). More than 80% of these programmes were developed in a multi-professional primary care practice (MPCP) or a healthcare centre, mostly in urban areas (58.3%). Some programmes had more than one funder (20.2%). The main financial contributor was the ARS (89.3%). All SMI programmes had a coordination structure, mainly with one coordinator (85.8%) who was trained in 69.7% of cases (Table 3). The coordinator was mainly a GP (34.7%), followed by a nurse (20.2%). The mean HCP number per programme was 8.1, among whom 5.2 were trained in self-management. The programme was rarely co-developed with patient associations (18.3%). When patients were part of the SMI team (26.7%), they were mainly trained in self-management (76.3%). Almost all SMI programmes worked with at least one local partner (98.4%; mean of 2.9 partners per programme). The main local partner was the hospital (19.3%). Lastly, 79% of SMI programmes covered only one topic, mainly diabetes (34.6%) (Table 4). Almost 42% of SMI programmes were open to patients with more than one disease, mainly adults (82.3%), including their relatives (91.8%). For 51.7% of SMI programmes, the estimated annual number of beneficiaries was lower than 30. The workshop modalities were mostly mixed, with group and individual activities, mainly in the face-to-face format (96.9%).

**Table 1.** General description of self-management interventions (group and personalized interventions), in France (13 regions), over the period 2010–2022

Variables	*n* (%)
**Type of self-management interventions (** ***n*** **= 4931)**	
Group intervention	4922 (99.8)
Personalized action	9 (0.2)
**PROGRAMMES**	
**Number by region (** ***n*** **= 4922)**	
Île-de-France	1072 (21.8)
Nouvelle-Aquitaine	599 (12.2)
Grand-Est	584 (11.9)
Auvergne-Rhône-Alpes	487 (9.9)
Hauts-de-France	400 (8.1)
Provence-Alpes-Côte d’Azur	392 (8.0)
Bretagne	378 (7.7)
Pays de la Loire	367 (7.5)
Bourgogne-Franche-Comté	210 (4.3)
Normandie	206 (4.2)
Centre-Val de Loire	178 (3.6)
Guadeloupe	36 (0.7)
Guyana	13 (0.3)
**Location (** ***n*** **= 4 922)**	
Hospital	4033 (82.0)
Primary care	889 (18.0)
**Period of authorization in years (** ***n*** **= 4 922), mean (SD)**	
Hospital (*n* = 4 033)	7.3 (4.5)
Primary care (*n* = 889)	6.6 (4.0)
**IMPLICATED ADMINISTRATIVE STRUCTURES (** ***n*** **= 889)**	
**In primary care practices (** ***n*** **= 271)**	
Multi-professional primary care practice	174 (64.2)
Healthcare centre	35 (12.9)
National health insurance fund for agricultural workers and farmers	32 (11.8)
Coordination Support Mechanism	21 (7.8)
Nursing-social cooperation institutions	7 (2.6)
Association of healthcare providers	2 (0.7)
**Outside primary care practices (** ***n*** **= 618)**	
Support associations for self-management interventions	157 (25.6)
National Health Insurance centre	107 (17.5)
Associative care structures^a^	78 (12.7)
Associations of non-primary care health providers	56 (9.1)
Clinical-social setting	53 (8.7)
Coordination support mechanism	46 (7.5)
Patient association	24 (3.9)
Mental health centres	15 (2.4)
Local government	15 (2.4)
Medical spas	12 (2.0)
Territorial and professional healthcare communities	9 (1.5)
Health and sports centres	8 (1.3)
Residential care facilities for dependent older adults	7 (1.1)
City-hospital networks	6 (1.0)
Foundations	5 (0.8)
Private health insurances	4 (0.6)
Home care	4 (0.6)
Regional union of primary care providers	4 (0.6)
Others^b^	3 (0.5)
*Missing data*	*5 (–)*

a Dialysis centres, non-multidisciplinary medical centres, nurse offices, nursing home care services.

b ‘Centre de Ressource, d’Expertise et de Performance Sportive’ (CREPS), ‘Centre de Coopération Internationale en Recherche Agronomique pour le Développement’ (CIRAD), and one programme in Bretagne identified with the term ‘association’.

**Table 2.** Description of the SMI programmes carried out in primary care practices in France (13 regions) over the period 2010–2022 (*n* = 127)

Variables	*n* (%)
**Programmes with authorization and/or declaration files**	
Multi-professional primary care practice	73 (57.5)
Healthcare centre	33 (25.9)
Coordination support mechanism	18 (14.2)
Association of healthcare providers	3 (2.4)
**Type of territory** ^a^	
Urban area	74 (58.3)
Rural area	53 (41.7)
**Programme duration in years, mean (SD)**	6.9 (3.9)
**Number of financial contributors**	
One	67 (79.8))
Two	15 (17.9)
Three	2 (2.3)
*Missing data*	*43 (–)*
**Financial contributors** ^b^	
Regional health agency	75 (89.3)
Primary care practices	14 (16.7)
Local government	12 (14.3)
National health insurance centre	1 (1.2)
Patients	1 (1.2)
*Missing data*	*43 (–)*

a According to the Insee classification.

(*∼https://www.insee.fr/fr/information/6439600#:∼:text=La%20grille%20communale%20de%20densit%C3%A9%20permet%20de%20classer%20les%20communes,commune%20est%20consid%C3%A9r%C3%A9e%20comme%20dense).*

b The total is >100% because each programme could be supported by more than one funder.

**Table 3.** Description of the SMI teams in primary care practices, in France (13 regions), over the period 2010–2022 (*n* = 127)

Variables	*n* (%)
**COORDINATION**	
**Coordination role**	
Yes	127 (100)
No	0 (0)
**Number of coordinators per programme**	
One	109 (85.8)
Two	17 (13.4)
Three	1 (0.8)
**Coordination training for the main coordinator**	
Yes	69 (69.7)
No	30 (30.3)
*Missing data*	28 (–)
**Occupation of the main trained coordinator** ^a^	
General practitioner	24 (34.7)
Nurse	14 (20.2)
Coordination support mechanism coordinator	11 (15.9)
Dietician	8 (11.6)
Nurse administrator	2 (2.9)
Multi-professional primary care practice coordinator	2 (2.9)
Physiotherapist	2 (2.9)
Podiatrist	2 (2.9)
Adapted physical activity teacher	1 (1.5)
Other specialist	1 (1.5)
Patient expert	1 (1.5)
Prevention manager	1 (1.5)
**SMI TEAM**	
**Number of healthcare professionals per programme, mean (SD)**	8.1 (5.5)
**Number of trained healthcare professionals per programme, mean (SD)**	5.2 (5.4)
**ASSOCIATION AND PATIENT INTERVENTION**	
**Co-developed with patient associations**	
Yes	19 (18.3)
No	85 (81.7)
*Missing data*	*23 (–)*
**Number of SMI teams with at least one patient member**	
Yes	31 (26.7)
No	85 (73.3)
*Missing data*	*11 (–)*
**Trained patient in self-management member in the SMI team** ^b^	
Yes	29 (76.3)
No	5 (23.7)
*Missing data*	*4 (–)*
**LOCAL PARTNERS**	
**SMI programme with at least one local partner**	
Yes	62 (98.4)
No	1 (1.6)
*Missing data*	*64 (–)*
**Number of local partners per programme** ^c^ **, mean (SD)**	2.9 (2.1)
*Missing data*	*64 (–)*
**Type of local partner** ^d^	
Hospital	35 (19.3)
Health theme expert	34 (18.8)
Support association of self-management intervention	29 (16.0)
Patient association	26 (14.5)
Clinical-social institution	20 (11.1)
Other primary care practices	11 (6.1)
Local government	10 (5.5)
French local health insurance fund	6 (3.3)
Health and sports centres	4 (2.2)
Private actors	3 (1.6)
Others^e^	3 (1.6)

SD: Standard deviation, SMI: Self-management interventions.

a Among the main coordinators trained in coordination *n* = 69.

b Among patients participating in SMI programmes *n* = 38.

c Among the SMI programmes with at least one local partner *n* = 62.

d Among all local partners *n* = 181.

e Student and other associations.

**Table 4.** Description of the SMI programmes in primary care practices, in France (13 regions), over the period 2010–2022 (*n* = 127)

Variables	*n* (%)
**PROGRAMME DETAILS**	
**Number of topics**	
One	100 (78.7)
Two	27 (21.3)
**Main topic**	
Diabetes	44 (34.6)
Diabetes and cardiovascular diseases	20 (15.6)
Cardiovascular diseases	13 (10.2)
Obesity	12 (9.5)
Respiratory diseases	11 (8.7)
Chronic disease combination^a^	10 (7.9)
Chronic pain	8 (6.3)
Neurodegenerative diseases	4 (3.2)
Cancers	2 (1.6)
Psychiatric diseases	1 (0.8)
Arrhythmic disorders	1 (0.8)
Rheumatic diseases	1 (0.8)
**Number of pathologies**	
One	74 (58.3)
Several	53 (41.7)
**Targeted patients**	
Adults	102 (82.3)
Children and/or teenagers	9 (7.2)
Both	13 (10.5)
*Missing data*	*3 (–)*
**Inclusion of relatives**	
Yes	89 (91.8)
No	8 (8.2)
*Missing data*	*30 (–)*
**Estimated annual number of beneficiaries**	
≤30	61 (51.7)
31–49	21 (17.8)
≥50	36 (30.5)
*Missing data*	*9 (–)*
**WORKSHOP FEATURES**	
**Workshop modalities**	
Group	49 (40.5)
Individual	1 (0.8)
Mixed (group and individual)	65 (53.7)
Group, individual options	6 (5.0)
*Missing data*	*6 (–)*
**Workshop format**	
Face to face	123 (96.9)
Mixed workshop (face to face and remote)	4 (3.1)
Remote workshop	0

a Combination of different chronic diseases for each programme (1 to 10): 1 (obesity and cardiovascular diseases); 2 (diabetes, heart failure, Parkinson’s disease, cancer, depressive disorders and asthma, and/or chronic obstructive pulmonary disease, COPD); 3 (multiple pathologies in the context of ageing without cognitive disorders); 4 (cardiovascular diseases, respiratory diseases, and type II diabetes); 5 (severe chronic kidney disease and primary nephrotic syndrome); 6 (ulcerative colitis and progressive Crohn’s disease); 7 (cardiovascular diseases, obesity and stress management); 8 (asthma, cardiovascular diseases, kidney failure, and type II diabetes); 9 (stroke, diabetes, kidney failure, neurological diseases, cancers, cardiovascular diseases, rheumatic diseases, digestive system diseases, respiratory diseases, and HIV/AIDS); and 10 (type II diabetes, cardiovascular diseases, and COPD.

## Discussion

The results of this study showed that in France, less than 20% of SMI programmes are carried out in primary care settings and that 5.5% (*n* = 271) of them are developed in PCPs. Moreover, they focus mostly on adults with one chronic disease, and they are followed by too few patients. They are mainly supported financially by the ARS; they have a large team, with a quasi-systematic coordination role, carried out by GPs and nurses. Although they are well integrated with the territorial partners (first the hospital), most programmes do not include a patient association partnership.

As previously shown in other European countries, and despite the recommendations to integrate SMI in primary care service routine, SMI programmes in primary care in France remains rare (Boerma *et al.*, 2015; Elissen *et al.*, 2013). Moreover, as their content focuses often on only one chronic disease, they are inadequate to meet the population’s needs. This result is in line with an international systematic review of 2024 showing that only 20% of SMI programmes in primary care focused on multimorbidity (Allory *et al.*, 2024). However, a retrospective cohort study on more than 400,000 people in England found that patients with multimorbidity accounted for more than half of GP consultations and 78.7% of their prescriptions (Cassell *et al.*, 2018). Moreover, a 2025 study by the Organization for Economic Co-operation and Development on 107,000 people from 19 countries showed that 54% of individuals older than 45 years followed in primary care had at least ≥2 chronic diseases (OECD, 2025). It seems that the recommendations by Rijken *et al.* in 2014 to develop a multimorbidity approach for SMI in primary care are still relevant, but have not been followed (Rijken *et al.*, 2014). Some qualitative studies with primary care professionals showed that self-management among people with multimorbidity is perceived ‘*as “chipping away”, using regular consultations, continuity and ongoing education to encourage change*’ (Kristensen *et al.*, 2017). Thus, the current SMI programmes may not be adapted to all patient needs and may hinder the development of self-management for people with multimorbidity. Based on these results, the French national health authority, which is not currently supporting the development of multimorbidity approaches in SMI, could encourage them in different ways (e.g. continuing professional development, financial incentives). Another solution could be to combine individual and collective self-management activities (Traynard and Gagnayre, 2013). Thus, the individual approach, which can be repetitive, during and after the SMI programme, can favour a more patient-centred approach, and the collective approach can help the development of soft skills. In any case, although SMI effectiveness on multimorbidity remains unclear, it seems a priority to work on new SMI models to meet the needs of patients with multimorbidity (Smith *et al.*, 2021).

In our study, the SMI programme coordinator, who is required in France, was mainly a GP or a nurse. This is in line with the 2014 study by Nolte *et al.* on chronic disease management in European health systems showing that in the majority of cases, SMIs are provided by GPs or trained nurses (Nolte *et al.*, 2014). However, in many European countries, it is uncommon to have nurses as SMI programme coordinators. For instance, Kringos *et al.* found that nurses provide health education in primary care only in 12/31 European countries (Kringos *et al.*, 2015). Our result could be explained by the fact that in France, SMI programmes in primary care are mainly developed at PCPs and that the nurses’ implication in care delivery and coordination is common in systems with multidisciplinary teams (Nolte, 2009). This involvement could be partially due to the new care organization in France that gives a bigger place to PCPs and includes more collaboration between HCPs. This leads to responsibility delegation and new competencies, especially for nurses (Suchier and Michel, 2021). In the last years, the implementation of more advanced roles for nurses (e.g. advanced practice nurses) is a sign of this evolution. Another reason of the nurses’ implication is the increasing gap between the significant population’s demand for managing chronic diseases and the insufficient supply of GPs in the healthcare system. This change is a great opportunity because nurses seem to be supported in their mission by the population and considered by the SMI team as an important educator, with a better understanding of psychosocial issues (Barreto *et al.*, 2019).

Among the PCPs that deliver SMIs, MPCPs and healthcare centres were the most important. In France, both structures need to have a health project validated by the ARS, but have a different organization. In a MPCP, HCPs are self-employed and work with other self-employed HCPs in the same territory (sometimes the same building), whereas in a healthcare centre, HCPs are employees and work in a building with other HCPs. In 2024, a qualitative study with different HCPs showed that in the context of chronic disease, a collaborative approach among SMI actors is one of the necessary skills for developing SMI programmes in primary care (Timmermans *et al.*, 2024). In addition, our study found that PCPs are developing many local partnerships, mainly with the hospital, as discussed by Cret (Cret *et al.*, 2022). Nevertheless, our result is in line with the perspectives by Boerma *et al.* who wrote that the cooperation and coordination between primary and secondary care can benefit from the creation of multidisciplinary teamwork and practices (Boerma *et al.*, 2015).

Lastly, our analysis highlights the limited involvement of patients in SMI development and delivery. The recommendation to better involve the patients in the SMI teams is not followed yet (Reynolds *et al.*. 2018). However, some key decisions have been recently taken in France to facilitate the patients’ engagement in SMI. Indeed, the national health authority wrote in 2020 a recommendation on how to support the engagement of the population at all levels of the healthcare system, including SMI delivery in primary care (Haute Autorité de Santé, 2020).

Our study confirms European data on self-management and gives a national perspective using centralized data and a standardized approach based on ARS data. Although not all ARS participated, the collected data were from 13 ARS that cover more than 85% of the French population. However, we cannot exclude a selection bias, although we think that data were missing at random for several programmes.

Another strength of our study is to have worked using a structured administrative form. As we did not find publications reporting similar data from elsewhere in Europe, it should encourage other European countries to systematically describe who offers SMI in the healthcare system.

A limitation of the study is that we lacked data especially on the coordination training for the coordinator and on the engagement of associations or patients in the SMI team. This indicates that when SMI teams start the SMI process, they are often not trained in coordination and in collaboration with patients and/or associations. To explain this lack of patient engagement, Ocloo *et al.* showed in a systematic review of reviews that the patients’ engagement in the healthcare system needs a whole system approach (Ocloo *et al.*, 2021). Indeed, both the individual level (especially attitudes and representations among patients and healthcare professionals) and the collective level (communication and working with communities, funding, and general support to engage people especially) need to be taken into account to improve patient engagement. This systematic review did not include any study on patient engagement in self-management in primary care. However, the authors stressed that organizational factors play a key role in patient engagement. Therefore, more studies are needed on patient engagement in self-management programmes in primary health care. Furthermore, the set of specifications was completed by the SMI teams at the SMI beginning and was not re-evaluated afterwards. As the mean programme duration was 6.9 years (3.9), the SMI team probably underwent some changes over time. Therefore, we may have described the facilitating factors to implement SMI programmes in PCPs, but not the facilitating factors to maintain and pursue them.

Based on our results, we propose the following recommendations to French national health authority to improve SMI practice and access in primary care. First, a multimorbidity approach should be encouraged in SMI programmes in primary care. Second, nurses should be seen and supported as the main educator in PCPs. Third, policymakers should support the development of MPCPs and healthcare centres. Fourth, they should give financial incentives to the centres that develop SMI programmes. Fifth, the involvement of patients in SMI development and delivery should be promoted, in accordance with the French health authority recommendations (Haute Autorité de Santé, 2020).

## Conclusion

In France, self-management programmes in PCPs are still rare, lack a multimorbidity approach, and are carried out mainly in PCPs with inter-professional collaboration. GPs and nurses are the main actors in SMI programmes, whereas patients are rarely involved in their development and delivery. A qualitative study could be conducted among SMI educational teams in PCPs in France to better identify the SMI strengths, obstacles, and areas of improvement in order to promote a better model of SMI practice in PCPs.

## Supporting information

Allory et al. supplementary material 1 Allory et al. supplementary material

Allory et al. supplementary material 2 Allory et al. supplementary material

## Supplementary material

The supplementary material for this article can be found at https://doi.org/10.1017/S1463423626100929.
