# Supplementary material for: Self-management interventions in primary care practices in France between 2010 and 2022: a descriptive national study
Source: Prim Health Care Res Dev. 2026 Mar 6;27:e34. doi: 10.1017/S1463423626100929 (PMC12979017; doi:10.1017/S1463423626100929)
Supplement: Allory et al. supplementary material 1 — Allory et al. supplementary material [file S1463423626100929sup001.docx]

# Supplementary material 1: Set of specifications following the appendix 2 of the 2010’s decree constituting the application for authorization of a Self-management intervention (SMI) programme to the Regional health agency

Available on line : https://www.legifrance.gouv.fr/loda/id/JORFTEXT000022664592/

## The SMI team

### Coordinator of the SMI programme:

- Name:
- Professional address:
- Occupation:
- Organization:
- Any training and/or experience in self-management intervention:
- For each team member, provide the same information in an appendix, together with a photocopy of the document(s) attesting to the team member(s)' expertise in therapeutic education.

### Patient in the team

- Does the team include one or more patients involved in implementing the program?
  Yes/No

### Health care organization hosting the programme

- Name:
- Address:
- Legal status:
- Opening hours:
- If the programme includes more than one host structure, provide the same information for each structure in an appendix.
- If the program involves more than one region, provide all the information relating to the team section in the appendix for each region.

## The SMI programme

- Title
- Was this an operational programme prior to the promulgation of the law of July 21, 2009 on hospital reform and patients, health and territories? Yes No. If yes, since which year.
  - Is this an operational programme after publication of the law of July 21, 2009? Yes No
- For programmes coordinated by a doctor or other healthcare professional:
  - Has the program been co-constructed with a patient association approved under Article L. 1114-1 of the French Public Health Code? Yes No. If yes, with which association?
  - Will this association be involved in the program? Yes No
- For programmes whose coordinator is a member of a patient association approved under article L. 1114-1 of the French Public Health Code:
  - Has the program been co-constructed with a medical team? Yes No. If yes, with which team?
  - Will this medical team be involved in the programme? Yes No
- Which health problem(s) does the program address?
  - At least one of the 30 long-term conditions exempting patients from co-payment (ALD 30), asthma or one or more rare diseases.
  - A regional priority. Please specify:
  - Other. Please specify:
- If possible, what is the estimated number of potential beneficiaries of the programme?
- Is the SMI programme coupled with an accompanying action as defined in article L. 1161-3? Yes No
- Attach a two-page maximum description of the programme in the appendix, ensuring that it includes all the elements in the programme box of the specifications.

## La coordination

- Briefly describe how coordination and information between programme participants will be ensured.
- Briefly describe the coordination and information arrangements with other parties involved in the patient's care, in particular the attending physician.

Ethics, confidentiality and deontology

- Attach a copy of the patient's information and consent documents for program entry.
- Briefly describe how the confidentiality of patient data (including computerized data, if applicable) is ensured, and how the patient's consent to enter the programme and consent to the transmission of data concerning him/her will be obtained.
- Where applicable, will the use of individual data give rise to a request for authorization from the CNIL? Yes No
- Please attach a copy of the confidentiality agreement to be signed by all participants.
- Enclose a copy of the code of conduct to be signed by all participants.

## Programme evaluation

- Briefly describe how and on what criterion(s) the annual program self-evaluation will be carried out.
- Briefly describe how the program will be evaluated every four years, and on the basis of which criteria.

## Transparency

- List any sources of funding for the program, specifying the amount for each source.
