# Supplementary figures and images for: Self-management interventions in primary care practices in France between 2010 and 2022: a descriptive national study
Source: Prim Health Care Res Dev. 2026 Mar 6;27:e34. doi: 10.1017/S1463423626100929 (PMC12979017; doi:10.1017/S1463423626100929)

# Supplementary material 2: Agreement of the University Hospital Ethics Committee


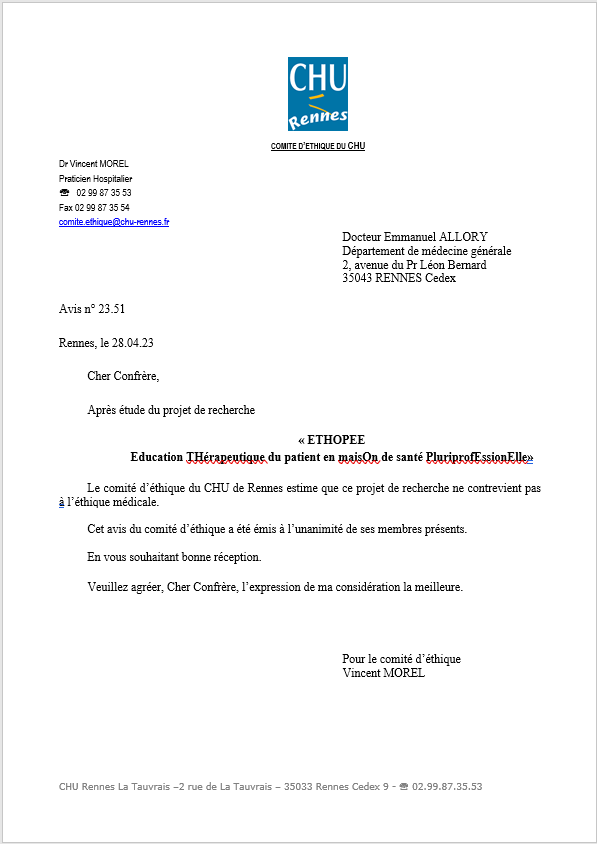

Supplement: Allory et al. supplementary material 2 — Allory et al. supplementary material [file S1463423626100929sup002.docx]
